# The Local Government Board's Opportunity

**Published:** 1914-12-26

**Authors:** 


### THE LOCAL GOVERNMENT BOARD'S OPPORTUNITY.

The Prestwich Board of Guardians at the outbreak of the war offered two wards in the Booth Hall Infirmary to the War Office for the treatment of wounded soldiers. The offer was gratefully accepted conditionally upon the sanction of the Local Government Board being received. At a recent meeting of the Guardians there was read a letter from the Local Government Board laying down three conditions which must be fulfilled before it will sanction any such arrangement in the case of a Poor-Law Infirmary. These conditions are, first, that a request must be received from a General Officer commanding or from the War Office; secondly, preference will be given to offers of a complete institution or of a complete block which, though it might be administered by the Guardians' staff, could be kept altogether distinct from the rest of the institution; and thirdly, the Board must be satisfied that the allocation of beds to wounded sailors or soldiers will in no way interfere with the accommodation required for the sick poor of the parish.

As regards the first and third of these conditions, no objection could possibly be raised. Naturally, as regards the first, the Guardians' offer is contingent upon a request from the military authorities for the utilisation of the accommodation offered, and as regards the third, no Guardians would offer beds which they could not spare without interfering with their ordinary work. We may, however, lay stress upon the fact, to which we have repeatedly drawn attention, that the best of our Poor-Law Infirmaries include a number of inmates who could be treated quite as well in less elaborate institutions, and that if a system of classification based upon medical requirements were applied, many beds would be available for the treatment of acute cases. The second condition is, however, open to more criticism. Many infirmaries which could give up, without difficulty, one or two wards for the reception of wounded or sick soldiers, might find it impossible to devote a whole block to their needs. Does there, indeed, seem to be any real necessity for this requirement? In most infirmaries a ward is a self-contained unit, and if it is necessary, as the Local Government Board would seem to suggest, to segregate the soldier from the rest of the inmates, may not this condition be as well met by the allocation of a single ward to his uses as by the allocation of a whole block?

The letter of the Local Government Board is remarkable, however, not for the conditions it lays down, but for the omission of any reference to the most important requirement of all—that the infirmary should be adequately equipped and staffed to deal with a number of acute medical and surgical cases in addition to its ordinary inmates. If all our Poor-Law infirmaries were administered on the lines of our general hospitals, this requirement would not, of course, be necessary; but we know as a matter of fact that such is not the case. The majority of them are staffed on the assumption that most of the cases are of a chronic character requiring little medical or nursing attention. There can be no other explanation of the vast discrepancy which exists between the staffing of the Poor-Law infirmaries and that of the voluntary hospitals. The assumption we know is, in the majority of cases, a false one, and the staff provided is altogether inadequate to carry out the work which already devolves upon it. This renders it the more impossible for it to deal efficiently with an additional number of acute cases. Not only is this the case, but many of our Poor-Law infirmaries have no *x*-ray apparatus or other facilities for special diagnostic investigations, are without a proper operating unit, and very little surgical work is carried out in them. Guardians, in their praiseworthy zeal to be of assistance, have often overlooked these points. It is therefore the first duty of the Local Government Board, before giving its sanction to any arrangement between Guardians and the War Office, to insist upon the provision of the necessary equipment and of a staff adequate as regards its numbers and expert knowledge to deal with the cases it is likely to receive. If the Local Government Board and the War Office take up this position, the result will be an improvement in the general efficiency of our infirmaries the good effect of which will be felt long after the war has ceased.

